# Diet-induced pre-diabetes slows cardiac conductance and promotes arrhythmogenesis

**DOI:** 10.1186/s12933-015-0246-8

**Published:** 2015-07-14

**Authors:** Lene Nygaard Axelsen, Kirstine Calloe, Thomas Hartig Braunstein, Mads Riemann, Johannes Pauli Hofgaard, Bo Liang, Christa Funch Jensen, Kristine Boisen Olsen, Emil D. Bartels, Ulrik Baandrup, Thomas Jespersen, Lars Bo Nielsen, Niels-Henrik Holstein-Rathlou, Morten Schak Nielsen

**Affiliations:** Department of Biomedical Sciences, Faculty of Health and Medical Sciences, The Danish National Research Foundation Centre for Cardiac Arrhythmia, University of Copenhagen, Blegdamsvej 3B, Copenhagen, N DK-2200 Denmark; Department of Veterinary Clinical and Animal Sciences, University of Copenhagen, Copenhagen, Denmark; Department of Biomedical Sciences, Faculty of Health and Medical Sciences, Core Facility for Integrated Microscopy, University of Copenhagen, Copenhagen, Denmark; Department of Forensic Medicine, Section of Forensic Pathology, University of Copenhagen, Copenhagen, Denmark; Department of Clinical Biochemistry, Rigshospitalet, Copenhagen, Denmark; Centre for Clinical Research, Vendsyssel Hospital/Department of Clinical Medicine, Aalborg University, Aalborg, Denmark

**Keywords:** Diabetes, Electrophysiology, Arrhythmias, Gap junctions, Conduction velocity

## Abstract

**Background:**

Type 2 diabetes is associated with abnormal electrical conduction and sudden cardiac death, but the pathogenic mechanism remains unknown. This study describes electrophysiological alterations in a diet-induced pre-diabetic rat model and examines the underlying mechanism.

**Methods:**

Sprague–Dawley rats were fed either high-fat diet and fructose water or normal chow and water for 6 weeks. The electrophysiological properties of the whole heart was analyzed by *in vivo* surface ECG recordings, as wells as *ex vivo* in Langendorff perfused hearts during baseline, ischemia and re-perfussion. Conduction velocity was examined in isolated tissue strips. Ion channel and gap junction conductances were analyzed by patch-clamp studies in isolated cardiomyocytes. Fibrosis was examined by Masson’s Trichrome staining and thin-layer chromatography was used to analyze cardiac lipid content. Connexin43 (Cx43) expression and distribution was examined by western blotting and immunofluorescence respectively.

**Results:**

Following 6 weeks of feeding, fructose-fat fed rats (FFFRs) showed QRS prolongation compared to controls (16.1 ± 0.51 (*n* = 6) vs. 14.7 ± 0.32 ms (*n* = 4), *p* < 0.05). Conduction velocity was slowed in FFFRs vs. controls (0.62 ± 0.02 (*n* = 13) vs. 0.79 ± 0.06 m/s (*n* = 11), *p* < 0.05) and Langendorff perfused FFFR hearts were more prone to ventricular fibrillation during reperfusion following ischemia (*p* < 0.05). The patch-clamp studies revealed no changes in Na^+^ or K^+^ currents, cell capacitance or gap junctional coupling. Cx43 expression was also unaltered in FFFRs, but immunofluorescence demonstrated an increased fraction of Cx43 localized at the intercalated discs in FFFRs compared to controls (78 ± 3.3 (*n* = 5) vs. 60 ± 4.2 % (*n* = 6), *p* < 0.01). No fibrosis was detected but FFFRs showed a significant increase in cardiac triglyceride content (1.93 ± 0.19 (*n* = 12) vs. 0.77 ± 0.13 nmol/mg (*n* = 12), *p* < 0.0001).

**Conclusion:**

Six weeks on a high fructose-fat diet cause electrophysiological changes, which leads to QRS prolongation, decreased conduction velocity and increased arrhythmogenesis during reperfusion. These alterations are not explained by altered gap junctional coupling, Na^+^, or K^+^ currents, differences in cell size or fibrosis.

## Background

Metabolic disorders such as type 2 diabetes and metabolic syndrome are associated with abnormal electrical conduction in the heart, increased risk of cardiac arrhythmias, and sudden cardiac death [1–3]. The underlying pathophysiology, however, remains to be fully elucidated. Early on, an abnormal cardiac tissue composition, such as non-homogenous interstitial fibrosis, was identified as a potential cause of enhanced susceptibility to arrhythmias in diabetes [4]. Subsequently, studies in streptozotocin (STZ) induced type 1 diabetic rats, revealed decreased conduction velocity in the heart at baseline, along with a decreased conduction reserve in response to uncoupling [5, 6]. In the ventricles, conduction velocity and conduction reserve are determined by the excitability of individual cardiomyocytes, intercellular electrical coupling through gap junctions, as well as obstacles to the propagation of action potentials such as fibrosis. For STZ induced diabetic rats, gap junction remodeling, seen as altered expression of the major ventricular gap junction protein connexin43 (Cx43) [7, 8], and/or Cx43 lateralization [6, 9] are proposed as a potential mechanism for their altered conduction.

In the setting of insulin resistance and type 2 diabetes, rats fed a high fat diet (HFD) for 15 weeks display increased infarct sizes along with an increased mortality rate following ischemia-reperfusion [10]. These findings were correlated with minor but non-significant changes in Cx43 phosphorylation. In addition, our laboratory has recently shown that myocardial impulse propagation is impaired in Zucker Diabetic Fatty (ZDF) rats (a genetic model of type 2 diabetes) [11]. In the ZDF rat, we detected a minor degree of Cx43 lateralization, but it seems insufficient to explain the observed conduction impairment. Therefore, we hypothesize that other mechanisms are involved in the development of conduction slowing in the diabetic heart. The amount of available data on this topic is, however, limited and further studies are needed to identify the complex mechanism of electrical instability in patients with metabolic disorders.

We have previously validated fructose-fat fed rats (FFFRs) as a useful diet-induced model of metabolic syndrome [12]. Rats fed a high fructose diet is known to develop a higher degree of hyperinsulinemia and glucose intolerance compared to high fat fed (HFF) rats [13], in contrast, the HFF rats become more obese. Combining the two diets gives a model that develops a combination of obesity, hypertriglyceridemia, and severe glucose intolerance, which makes the FFFR model similar to the phenotype of metabolic syndrome seen in humans [12]. After just 6 weeks of feeding, the FFFRs display increased fasting blood glucose and a significantly impaired glucose tolerance [12]. This makes the FFFR a pre-diabetic model at 6 weeks of feeding. Intramyocardial lipid accumulation is extensive at 6 weeks of high-fructose-fat feeding, but cardiac hemodynamics remain uncompromised during unstressed conditions for up to 56 weeks of feeding [12]. Nevertheless, FFFRs develop cardiac hypertrophy within 18 weeks of feeding, as well as an increased vulnerability to ischemia-reperfusion following 56 weeks on the fructose-fat diet [12]. This study describes electrophysiological alterations in the hearts of FFFRs and aims at identifying the underlying cellular mechanisms. In order to avoid interactions from long-term diabetic complications, the FFFR hearts were evaluated in the early pre-diabetic state following 6 weeks of feeding.

## Methods

### Animal model

Animal studies were performed according to the Guide for the Care and Use of Laboratory Animals, published by the US National Institutes of Health (NIH publication No. 85–23, revised 1996) and approved by the Animal Experiments Inspectorate of the Danish Ministry of Justice. Four-week-old male Sprague–Dawley rats (Taconic, Ll. Skensved, Denmark) were randomly stratified into two groups. One group (controls) received unlimited normal chow (13 kcal % fat, Altromin 1319, Brogaarden, Lynge, Denmark) and water, and the other group, fructose-fat fed rats (FFFR), received unlimited high-fat diet (60 kcal % saturated fat, D12492, Research Diets, New Brunswick, USA) and 10 % fructose (F0127, Sigma-Aldrich, Brøndby, Denmark) in the drinking water as previously described [12]. To avoid bacterial growth in the drinking water, citric acid was added to give a pH of 3.6 in both control and fructose water. Rats were kept on this special diet for 6 weeks before entering the experiments described below.

### Blood samples, fasting glucose and insulin measurements

Rats fasted for 16 h were gently restrained and their fasting blood glucose was measured on a (~2 μL) tail blood sample using a FreeStyle Lite analyzer (Abbott, Copenhagen, Denmark). Subsequently, a sample of tongue blood (~300 μl) was collected and transferred to heparin containing micro tubes (VWR, Herlev, Denmark). The samples were centrifuged at 1500 g for 15 min at 4 °C. Plasma was collected and stored at −80 °C for later analysis.

Plasma insulin was measured with ELISA (cat no. EIA2048, DRG International, Marburg, Germany) according to the manufactures instructions.

### *In vivo* ECG recordings

Seven needle electrodes were inserted subcutaneously into lightly anesthetized (1.5 % isoflurane in O_2_) rats. Four electrodes were connected to the limbs, while the remaining electrodes were placed precordially. ECG’s were obtained with an electronic recorder (GE MAC 800, GE Healthcare, Milwaukee, WI). ECG recordings were transferred to a central database (GE Muse Cardiology Information System, GE Healthcare, Milwaukee, WI) and analyzed manually in a blinded fashion using an electronic ruler. Measurements included P-wave duration and amplitude, QRS-complex duration and amplitude as well as QT-interval and corrected QT-interval (QTc) using formulas proposed by Bazett and Kmecova [14]. The QRS width was measured from the beginning of the Q-wave to the negative peak of the S-wave. QT was measured from the beginning of the Q-wave to the point where the downslope of R’ return to the isoelectric line.

### Conduction velocity and contractility of ventricular strips

Conduction velocity and contractility was measured in tissue strips from the free wall of the right ventricle as previously described for atrial tissue [15]. In short, hearts were explanted from rats anesthetized with 5 % isoflurane in 35 % O_2_/N_2_. Tissue strips were mounted in a 1 ml chamber and perfused with 35 °C, 100 % O_2_ bubbled Tyrode’s buffer (mM: 136 NaCl, 4 KCl, 0.8 MgCl_2_, 1.8 CaCl_2_, 5 HEPES, 5 MES, 10 Glucose, pH 7.3) at a flow rate of 2 ml/min. The tissue was paced with a unipolar stimulation electrode at 1 Hz, impulse duration 0.5 ms and double threshold voltage (Master-8 stimulator, A.M.P.I., Jerusalem, Israel). Conduction velocity was measured using two extracellular microelectrodes (Platinium/Iridium (PI20030.5B10), Micro Probe Inc., Gaithersburg, USA) placed on the longitudinal axis of the tissue strip. Time of local activation under the first and second microelectrode was determined as the time of minimum dU/dt by custom written MatLab script. Conduction velocity was calculated as the inter-electrode distance divided by the inter-electrode delay. Force was recorded continuously using the isometric force-transducer. Muscle length was adjusted to the level where contractions under control conditions were 50 % of maximal. Following a 15 min resting period, conduction velocity was measured over a 20 min period and developed force and passive tension were analyzed and calculated by the custom written MatLab script.

### Ischemia-reperfusion study

Rats were anaesthetized with 5 % isoflurane in 35 % O_2_/N_2_ and the aorta was cannulated as previously described [16]. The hearts were transferred to a perfusion apparatus (Hugo Sachs Elektronik – Harvard Appartus GmbH, March-Hugstetten, Germany) and perfused in the Langendorff mode with modified Krebs-Henseleit solution (mM: 118 NaCl, 4.7 KCl, 1.75 CaCl_2_, 1.2 KH_2_PO_4_, 1.2 Mg_2_SO_4_, 24.9 NaHCO_3_, 11.0 glucose) under continuously bubbling with carbogen (95 % O_2_/5 % CO_2_) and a constant perfusion pressure of 80 mmHg. A fluid filled balloon (size 5) (Hugo Sachs Elektronik – Harvard Appartus GmbH, March-Hugstetten, Germany) was inserted through an incision in the left auricle into the left ventricle to allow measurements of left ventricular pressure (LVP). The volume of the balloon was adjusted to give an end-diastolic pressure of approximately 5 mmHg. Subsequently, a ligature was placed around the left anterior descending coronary artery (LAD), enabling induction of ischemia. The experiment progressed as 30 min of normal perfusion (baseline), 30 min of LAD occlusion, and 60 min of re-perfusion. Langendorff studies were recorded and evaluated using iox2 version 2.4.2.6 software (emka TECHNOLOGIES, Paris, France).

### Area at risk and infarct size

At the end of Langendorff experiments the LAD ligature was re-clamped and the heart perfused with Evans Blue dye (0.1 %) (SIGMA, E2129) to evaluate the area at risk of infarction. Subsequently, the heart was sliced into ~2 mm thick vertical slices and incubated in 2,3,5-triphenyltetrazoliumchloride (TTC, SIGMA T8877) 10 mg/ml in 0.1 M phosphate buffer (pH 7.4) for 10 min at 37 °C. Subsequently, the tissue slices were washed 3 times in MilliQ water and transferred to 4 % formalin overnight. Finally, the tissue slices were weighed and scanned on both sides at 1200 DPI to analyze the infarct size. Pictures were analyzed in a blinded fashion using ImageJ software.

### Arrhythmia analysis

ECG recordings during Langendorff experiments were obtained using a 6-lead Einthoven ECG recording system (Hugo Sachs Elektronik – Harvard Apparatus GmbH, March-Hugstetten, Germany) and analyzed using ecgAUTO v2.8.1.26 software (emka TECHNOLOGIES, Paris, France). In short, individual ECG libraries were generated for each experiment. QRS and QT duration were analyzed and the number of extra systoles (also known as a ventricular premature complex), as well as the number, morphology and duration of each episode of arrhythmia determined. In general, the arrhythmia analysis complies with the updated Lambeth conventions [17]. Episodes of VT were defined as at least 4 consecutive ventricular complexes, however, we did not distinguish between monomorphic, polymorphic, or Torsades de pointes VTs. VF was defined as a continuous entry for which individual QRS complexes could no longer be distinguished from each other. In regard to QT duration, the end of the T-wave was set as the point where the downslope returned to the isoelectric line.

### Isolation of cardiomyocytes

Rats were anesthetized by 5 % isoflurane in 35 % O_2_/N_2_ and the heart perfused in a custom made perfusion system at a constant pressure of 60 mmHg. The heart was perfused with Tyrode’s solution (mM: 136 NaCl, 4 KCl, 5 HEPES, 5 MES, 0.8 MgCl_2_, 1.8 CaCl_2_, 10 glucose, pH 7.4) for 5 min followed by 2 min perfusion with Ca^2+^ free Tyrode’s solution and 2 min with potassium-gluconate buffer (mM: 20 NaCl, 120 potassium gluconate, 1 MgCl_2_, 10 HEPES, 10 glucose, pH 7.4). Subsequently, the heart was perfused with potassium-gluconate buffer including collagenase (140–165 U/ml) (Type 2 from Worthington) until the heart was digested (approximately 20–25 min). The ventricles were then sliced into small pieces, placed in collagenase buffer and bubbled with 100 % O_2_ till the tissue was dissolved. The solution was filtered and left to settle for 10 min. Cardiomyocytes used for measurements of gap junction conductivity was gradually added Ca^2+^ to give a final concentration of 0.75 mM.

### Gap junction coupling

Intercellular coupling was measured in ventricular cell pairs at room temperature using the dual whole-cell patch-clamp method as previously described [18]. Cells were voltage clamped at −10 mV, and a 1 s −10 mV pulse was applied to one cell at 0.1 Hz. Data was analyzed using Cellworks Reader (NPI Electronic). The average intercellular conductance during a 90 s interval following patch break was calculated as the resulting current deflection in the passive cell (nonpulsed) divided by the transjunctional voltage difference using a custom-made MatLab script.

### Na^+^-channel activity

Whole-cell sodium currents (I_Na_) were recorded at room temperature on ventricular myocytes. The internal solution consisted of 5 mM NaCl, 135 mM CsF, 10 mM EGTA, 5 mM MgATP and 5 mM HEPES (pH 7.2 with CsOH) and the low-sodium external solution consisted of 20 mM NaCl, 1 mM CaCl_2_, 1 mM MgCl_2_, 0.1 mM CdCl_2_, 20 mM HEPES, 117.5 mM CsCl and 11 mM glucose (pH 7.4 with CsOH). To characterize the voltage dependence of the peak sodium current, single myocytes were held at −120 mV, and 200 ms voltage steps were applied from −80 to +15 mV in 5 mV increments. Interval between each steps was 3 s. Measurements were made with pClamp10 software and a MultiClamp 700B amplifier sampling at 20 kHz and filtering at 5 kHz (Molecular Devices, Axon Instruments, Sunnyvale, USA). Borosilicate glass pipettes were pulled on a DPZ-Universal puller (Zeitz Instruments, Martinsried, Germany). The pipettes had a resistance of 1.5–2.5 MΩ when filled with intracellular solution. The series resistance recorded in the whole-cell configuration was 2–5 MΩ and was compensated (80 %).

### K-channel activity

K^+^-channel voltage-clamp recordings were made using an EPC7 amplifier (HEKA Elektronik, Lambrecht/Pfalz, Germany) and digitized with a Digidata 1440A converter (Axon Instruments, Molecular Devices, Sunnyvale, California). pClamp10 software was used for data acquisition (Axon Instruments, Molecular Devices, Sunnyvale, California). Patch pipettes were fabricated from borosilicate glass capillaries (GC150F, Harvard Apparatus, Edenbridge, UK) using a gravity puller PIP5 puller (HEKA, Lambrecht/Pfalz, Germany) and the pipette resistance ranged from 1 to 3 MΩ. The cardiomyocytes were superfused with a HEPES buffer (mM: 126 NaCl, 5.4 KCl, 1.0 MgCl_2_, 2.0 CaCl_2_, 10 HEPES and 11 glucose, pH adjusted to 7.4 with NaOH and 300 μM Cd to block ICaL). The patch pipette solution had the following composition (in mM): 90 K-aspartate, 30 KCl, 5.5 glucose, 1.0 MgCl_2_, 5 EGTA, 5 MgATP, 5 HEPES, 10 NaCl, pH 7.2 with KOH. The experiments were performed at 37 °C. After a whole-cell patch was established, cell capacitance was measured by applying −5 mV voltage steps. From a holding potential of −80 mV, sodium currents were inactivated by a 10 ms step to −40 mV and potassium currents were activated by a step protocol ranging from −120 to 40 mV for 2 s. A voltage–current relationship of the initial part of the step protocol is shown. Compensation of series resistance to 60–70 % was applied to minimize voltage errors. All analog signals were acquired at 10–50 kHz and filtered at 4–6 kHz. All electrophysiological experiments were blinded during acquisition and data analysis.

### Plasma and cardiac lipid measurements

Enzymatic kits were used for measurements of free fatty acids (Wako NEFA C kit, TriChem Aps, Frederikssund, Denmark), cholesterol (CHOD-PAP; Roche Applied Science), and free glycerol and triglycerides (TR0100, serum triglyceride determination kit, Sigma) in plasma samples. Cardiac lipid content was measured by thin-layer chromatography (TLC) as previously described [19]. All samples were analyzed in duplicate on separate TLC plates and quantified by digital image analysis using the ImageJ software.

### Fixation of hearts for fibrosis and Cx43 staining

Rats were anaesthetized by 5 % isoflurane in 35 % O_2_/N_2_ and the hearts removed, cannulated and connected to a homemade perfusion system as described for isolation of cardiomyocytes. The hearts were perfused at 60 mmHg with modified Krebs-Henseleit solution for 2 min followed by 10 ml of 2 % paraformaldehyde (PFA) dissolved in PBS pH 7.2. The hearts were immersed in 2 % PFA solution and stored at 4 °C overnight. The fixed hearts were subsequently stored in 0.5 % PFA at 4 °C until staining.

### Immunofluorescence

Fixated hearts were coated in paraffin and cut in 12 μm thick slices. Slices of right ventricular tissue were deparaffinized, permeabilized and incubated with the primary antibodies anti-Cx43 (C6219, Sigma-Aldrich, St. Louis, MO, 1:1000) and anti- N-cadherin (C3865 Sigma-Aldrich, St. Louis, MO, 1:500) and the appropriate secondary ALEXA conjugated antibodies (Life Technologies, Carlsbad, CA) as previously described [11]. Slices were imaged using a laser confocal microscope (Zeiss LSM 780, Carl Zeiss, Jena, Germany) and a 63x Oil, NA 1.4 objective.

Amounts of total Cx43 and Cx43 not localized within 3 μm of N-Cadherin (regarded as Cx43 not localized in intercalated discs), were analyzed in a blinded fashion.

### Histomorphometric analysis

Sections of fixed ventricular tissue were in coated in paraffin and processed for Masson’s Trichrome staining and histological examination as previously described [11]. The analysis was made in a blinded fashion.

### Western blotting

Hearts were explanted from isoflurane anesthetized rats and homogenized as previously described [20]. Ten percent Bis-Tris gels and MOPS running buffer were used and the proteins were blotted onto nitrocellulose membranes (all from Life technologies, Nærum, Denmark). Anti-Cx43 (C6219) 1:50.000, anti-β-tubulin (T0198) 1:2000 (both from Sigma-Aldrich, Brøndby, Denmark), anti-mouse (IRdye680RD) 1:10.000, and anti-rabbit (IRdye800CW) 1:10.000 (LI-COR Biosciences, Cambridge, UK) were used and membranes analyzed using Odyssey CLx Infrared Imaging System (LI-COR Biosciences, Cambridge, UK).

### Statistical analysis

All data are presented as mean ± standard error of the mean (SEM). Statistical analysis was performed using GraphPad Prism 5 (GraphPad Software,Inc. La Jolla, CA) or SAS 9.2 (SAS Institute, Cary, NC). For single parameters, differences between groups were generally analyzed using an unpaired Students *t*-test. For a subgroup of data sets, variances were significantly different between groups. In these cases, an unpaired *t*-test with Welch’s correction was performed. The ECG and Langendorff data were analyzed using mixed models with repeated measures in PROC MIXED, followed by Duncan’s new multiple range test. *p* < 0.05 was considered statistically significant.

## Results

### General characteristics of FFFR and control rats

During 6 weeks of feeding, FFFRs developed increased body mass compared to control fed rats, whereas there heart weight remained unaffected (Table 1). In addition, FFFRs increased their fasting blood glucose and insulin levels. The plasma triglyceride level was also increased and the cardiac triglyceride content was 2.5 times higher in FFFRs compared to control fed rats (Table 1). Besides the increase in insulin levels, these general characteristics of FFFRs are consistent with what we have previously published [12]. In our previous study, however, hyperinsulinemia was not significant at 6 weeks of feeding, but presented between 6 and 12 weeks of feeding.

**Table 1:** General characteristics of fructose-fat fed rats (FFFR) and control rats

	FFFR	*n*	Control	*n*	*P*-value
Body mass (g)	393 ± 5.9	44	366 ± 4.9	44	<0.001
Heart weight (g)	1.16 ± 0.04	11	1.10 ± 0.06	11	ns
Fasting blood glucose (mM)	4.2 ± 0.10	44	3.8 ± 0.08	44	<0.01
Fasting insulin (pmol/L)	98.8 ± 17	15	35.0 ± 5.9	11	<0.01
Fasting plasma cholesterol (mM)	2.1 ± 0.12	12	2.2 ± 0.08	12	ns
Fasting plasma glycerol (mM)	0.30 ± 0.02	12	0.25 ± 0.03	12	ns
Fasting plasma triglyceride (mM)	1.2 ± 0.15	12	0.72 ± 0.06	12	<0.05^a^
Fasting plasma FFA (mM)	0.32 ± 0.02	12	0.38 ± 0.03	12	ns
Cardiac cholesterol (nmol/mg ww)	3.3 ± 0.23	12	2.8 ± 0.23	12	ns
Cardiac triglyceride (nmol/mg ww)	1.9 ± 0.19	12	0.77 ± 0.13	12	<0.0001

Data are presented as mean ± SEM

*FFA* free fatty acids, *ww* wet weight, *NS* non-significant

^a^an unpaired *t*-test with Welch’s correction was performed, due to significantly different variances in the two groups

### FFFRs display QRS prolongation *in vivo*

**Fig. 1:**
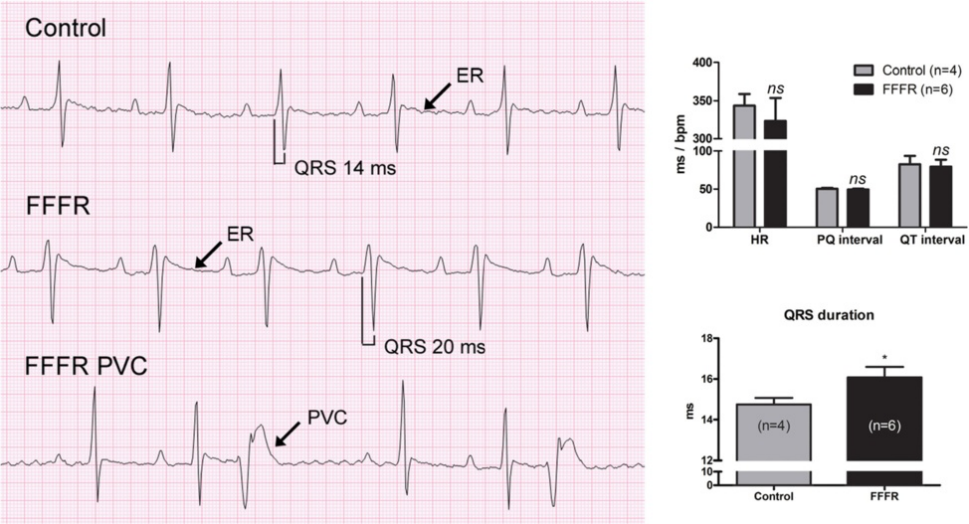
*In vivo* surface electrocardiogram (ECG) analysis from lightly anesthetized rats. Representative recordings from a control, a fructose-fat fed rat (FFFR) and a FFFR with premature ventricular complexes (PVCs) corresponding to a trigemini is shown on the left side. The average heart rate (HR), PQ and QT interval, as well as QRS duration is shown to the right. In rodents, there is no clearly demarcated T-wave as seen in larger mammals. The end of the repolarization was therefore determined manually, as the point where the slope from R’ (second R-wave) crosses the isoelectric line. This point is marked ER (end of repolarization) and used as the end of the measured QT interval. The data analysis was conducted in a blinded fashion and data are presented as mean ± SEM, *p < 0.05

*In vivo* ECG recordings from rats in light isoflurane anesthesia (Fig. 1) revealed that FFFRs had prolonged QRS duration compared to control rats (*p* = 0.017). In contrast, heart rate, P-wave duration and amplitude (P-wave data not shown), PQ, and QT intervals were not significantly different between the two groups (Fig. 1). QTc-Bazett intervals were 198 ± 27 ms and 184 ± 24 ms in control and FFFR hearts respectively (*p* = 0.7) and QTc-Kmecova [14] intervals were 76.7 ± 11 ms and 71.3 ± 9 ms respectively (*p* = 0.7). So in general, no difference was found in QT or QTc intervals irrespective of the QT correction formula used.

In addition to the prolonged QRS duration, premature ventricular complexes (PVCs) in sustained trigeminy (two sinus beats followed by a PVC) were observed in one of the six FFFRs (Fig. 1, lower left panel). None of the control rats showed any spontaneous arrhythmias.

### Conduction velocity is decreased in FFFRs

**Fig. 2:**
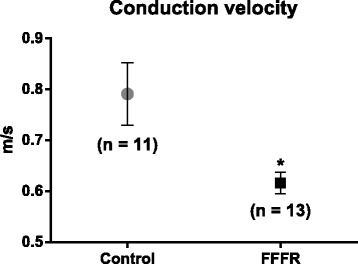
Ventricular conduction velocity. Conduction velocity was measured in the longitudinal direction of right ventricular strips from control and fructose-fat fed rats (FFFR). Data are presented as mean ± SEM, *p < 0.05

QRS prolongation represents a conductance abnormality, which may be explained by decreased conductance velocity in the ventricles. In support of this, measurements of impulse propagation in tissue strips from the right ventricle revealed a decreased conduction velocity in FFFRs compared to control rats (0.62 ± 0.02 vs 0.79 ± 0.06 m/s, *p* = 0.02) (Fig. 2). At the same time, developed force (119 ± 15 vs. 155 ± 24 mg/mm^2^, *p* = 0.21) and diastolic force (141 ± 22 vs. 202 ± 34 mg/mm^2^, *p* = 0.13) were not significantly different between tissue strips from FFFRs and controls, respectively.

### FFFRs and control rats respond differently to ischemia-reperfusion

To examine if the observed conductance abnormalities predispose FFFRs to cardiac arrhythmias, we evaluated both functional and electrophysiological characteristics of Langendorff perfused hearts, before, during and following 30 min of LAD occlusion. There were no statistically significant differences in either area at risk (27.1 ± 1.4 % and 27.5 ± 2.1 %, *p* = 0.87, for control and FFFR respectively) or infarct size (11.5 ± 1.6 % and 10.6 ± 0.92 %, *p* = 0.66, for control and FFFR respectively) following ischemia-reperfusion.

**Fig. 3:**
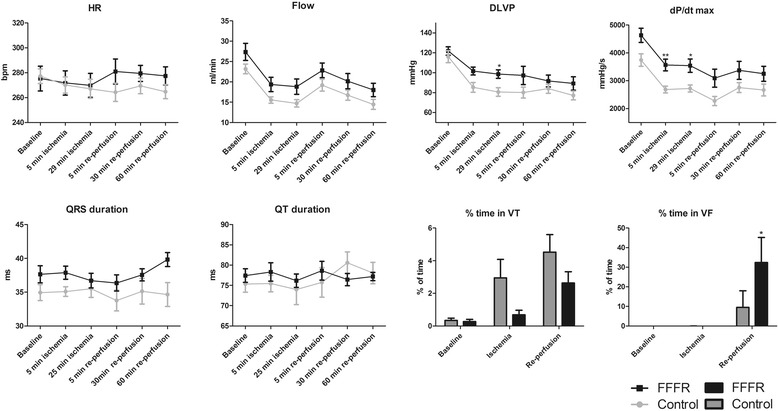
Functional and electrophysiological data for Langendroff perfused hearts. Data are presented from control and fructose-fat fed rats (FFFR), during baseline, 30 min of left anterior descending coronary artery (LAD) occlusion and 60 min of re-perfusion. HR: heart rate. DLVP: developed left ventricular pressure. dP/dt max: maximum change in pressure over time during systole. VT: ventricular tachycardia. VF: ventricular fibrillation. Data are presented as mean ± SEM, * *p* < 0.05, ** *p* < 0.001. *n* = 11 for both groups

As shown in Fig. 3, heart rate (HR) was not significantly different between the two groups. There was no statistically significant interaction term between group and time for flow, but the flow did change significantly with time (*p* < 0.0001). In addition, the flow was slightly higher in hearts from FFFRs compared to controls throughout the perfusion period, although the difference did not reach statistical significance (*p* = 0.055). Developed left ventricular pressure (DLVP) was not different between the two groups at baseline, the FFFR group, however, maintained a higher DLVP during ischemia (*p* < 0.05 between the two groups and *p* < 0.05 for the interaction between group and time). For the maximum increase in pressure over time during systole (dP/dt max) there was no significant interaction term between group and time. dP/dt max did, however, change significantly with time (*p* < 0.0001) and it was significantly higher in the FFFRs compared to controls (*p* < 0.01). Finally, the maximum decrease in pressure over time during diastole (dP/dt min) showed a significant interaction between group and time (*p* < 0.01) (data not shown).

For the electrophysiological parameters the mixed models ANOVA showed no interaction between group and time for QRS duration, which means that the effect of ischemia and re-perfusion on QRS length is not significant different between control and FFFR hearts. Though, looking at the entire perfusion period (baseline, ischemia and re-perfusion) the QRS complex was significantly prolonged in FFFRs compared to controls (*p* < 0.001). In contrast, we did not detect any significant differences in QT duration between the two groups and the QT interval was not significantly changed by either ischemia or re-perfusion. When evaluating the functional and electrophysiological data, it must be noted that the data only include observations from hearts in sinus rhythm. During the ischemic period, ten of eleven control hearts and six of eleven FFFR hearts had episodes of ventricular tachycardia (VT), whereas all hearts in both groups developed VT episodes during reperfusion. As seen in Fig. 3, there was a tendency towards the control hearts being in VT for a longer period of time than the FFFR hearts, however, it did not reach statistically significance (*p* = 0.07). Episodes of non-sustained ventricular fibrillation (VF) were also observed in both groups during both ischemia and reperfusion. Sustained VF was, however, only observed during reperfusion and occurred in one of eleven control hearts and five of eleven FFFR hearts. Summarizing episodes of sustained and non-sustained VF in the reperfusion period, FFFR hearts were significantly more prone to VF than control hearts (*p* < 0.05) (Fig. 3).

### Ion currents and gap junction conductance are unaltered in FFFRs

**Fig. 4:**
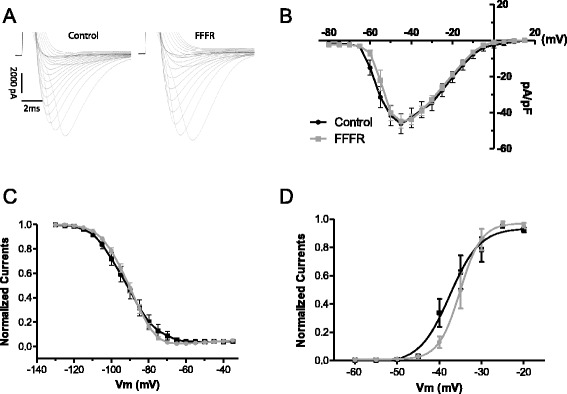
Whole-cell voltage-clamp Na^+^ current recordings in acutely isolated ventricular cardiomyocytes. **a** Representative sodium current traces following depolarisations from – 80 to 5 mV increments from both controls and fructose-fat fed rats (FFFR). **b** Current–voltage relationship of the peak sodium current normalised to cell capacitance. **c** Steady-state inactivation of the transient sodium current. **d** Steady-state activation of the sodium current. Data are based on 14 single cell recordings for both groups originating from 6 control and 4 FFFR hearts. Data are presented as mean ± SEM

The observed changes in cardiac conductance may be caused by changes in ion currents, decreased gap junction conductance, or altered tissue architecture in the form of changes in size of the individual cardiomyocytes or development of cardiac fibrosis. We used whole-cell voltage-clamp recordings of isolated ventricular cardiomyocytes to analyze sodium and potassium currents, gap junction conductance, as well as cell capacitance. Representative sodium current traces are shown in Fig. 4 along with the measured sodium current parameters. The peak current density as a function of voltage normalized to cell capacitance was unaltered between the two groups (Fig. 4b) and so was the steady state inactivation (V_½_ = −91.89 ± 2.33 mV vs −91.14 ± 1.09 mV, in controls and FFFRs respectively) (Fig. 4c) and the steady-state activation (V_½_ = −33.24 ± 0.93 mV vs −34.18 ± 2.89 mV, in controls and FFFRs respectively) (Fig. 4d). The late Na + current (I_NaL_) at −40 mV (measured as the mean current between 150 and 200 ms) also remained unchanged between the two groups (−2.14 ± 0.17 and −2.32 ± 0.21 pA/pF in controls and FFFRs respectively) (data not shown).

**Fig. 5:**
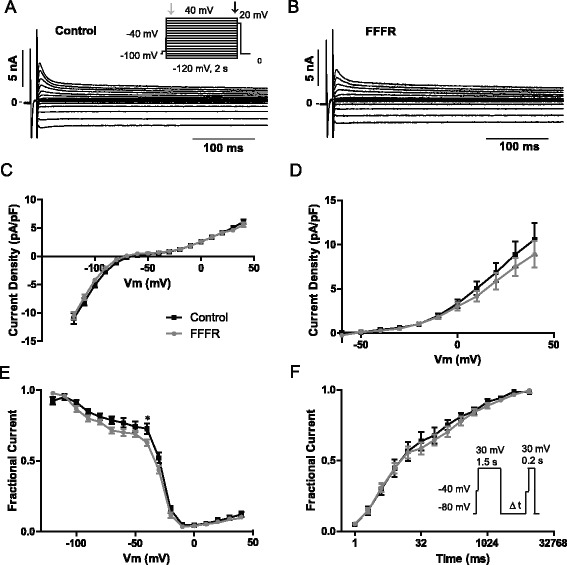
Whole-cell voltage-clamp K^+^ current recordings in acutely isolated ventricular cardiomyocytes. Representative recordings from control (**a**) and Fructose-fat fed rat (FFFR) (**b**) ventricular cardiomyocytes. **c** Sustained current density measured at the end of the voltage protocol (as indicated by the black arrow on the inserted protocol) as a function of voltage. (Control cells *n* = 17, FFFR cells *n* = 19). **d** Transient current density determined as the peak current density (measured at the start of the voltage protocol as indicated by the grey arrow) subtracted sustained current density. Control cells *n* = 17, FFFR cells *n* = 19. **e** Steady state inactivating control. Currents recorded at the +20 mV were normalized to maximal current, *n* = 16, FFFR, *n* = 19. **f** Time dependent recovery from inactivation was determined by a two-step protocol with increasing interpulse intervals, control *n* = 11, FFFR *n* = 19. Data are based on cells from 5 FFFR and 6 control hearts and presented as mean ± SEM, *p < 0.05

Representative potassium current recordings are shown in Fig. 5a (control) and b (FFFR). The sustained currents were measured at the end of the protocol and shown as a function of voltage in Fig. 5c. The inward component represents the inward rectifier current, I_K1_ and the outward current predominantly reflects sustained outward potassium currents, such as I_SS_, and no significant differences were observed between control and FFFR cardiomyocytes. The amplitude of the transient outward potassium currents, I_to_, was determined by subtracting the sustained currents from the peak current as shown in Fig. 5d. To further dissect the currents, steady-state inactivation was addressed as previously described by Himmel et al. [21]. Fig. 5e shows normalized currents recorded at the +20 mV tail step on the protocol shown as an insert in panel A. The voltage range from approximately −120 to −40 mV has previously been ascribed to a delayed rectifying current, whereas the current in the range from −40 to −10 mV has been ascribed to I_to_ and the remaining current to a sustained component such as I_SS_ [21]. I_to_ can be further divided into a current with a slow time dependent recovery from inactivation, I_to,s_, and a current component with a fast recovery from inactivation, I_to,f_. I_to,f_ down-regulation has repeatedly been associated with diseases such as heart failure and diabetic cardiomyopathy [22, 23] and as the data in Fig. 5d suggests a tendency toward reduced I_to_, we investigated if the contribution from I_to,f_ and I_to,s_ were affected in the FFFRs. Currents were elicited by a two-step protocol as shown in Fig. 5f and recovered currents were plotted as a function of interpulse-interval but no significant changes were observed between cardiomyocytes from controls and FFFRs.

**Fig. 6:**
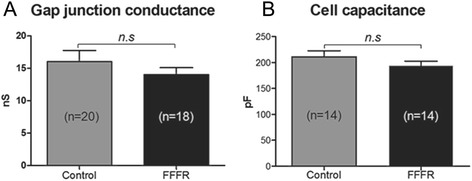
Gap junction conduction and cell capacitance. **a** Gap junction conduction measured by dual-clamp recordings on isolated cardiomyocyte cell pairs from 5 control and 4 fructose-fat fed rats (FFFR). **b** Cell capacitance of individual cardiomyocytes. Data are presented as mean ± SEM

Gap junction conductance was examined by dual-clamp recordings on ventricular cell pairs (Fig. 6a) but no statistical significant change in gap junction conductance was observed between the two groups (*p* = 0.33). Finally, cell capacitance, examined on individual ventricular cardiomyocytes, also remained unaltered between cells from control and FFFR hearts (Fig. 6b). This indicates that the sizes of the individual cardiomyocytes are similar between control and FFFR hearts.

### Connexin43 expression and localization

**Fig. 7:**
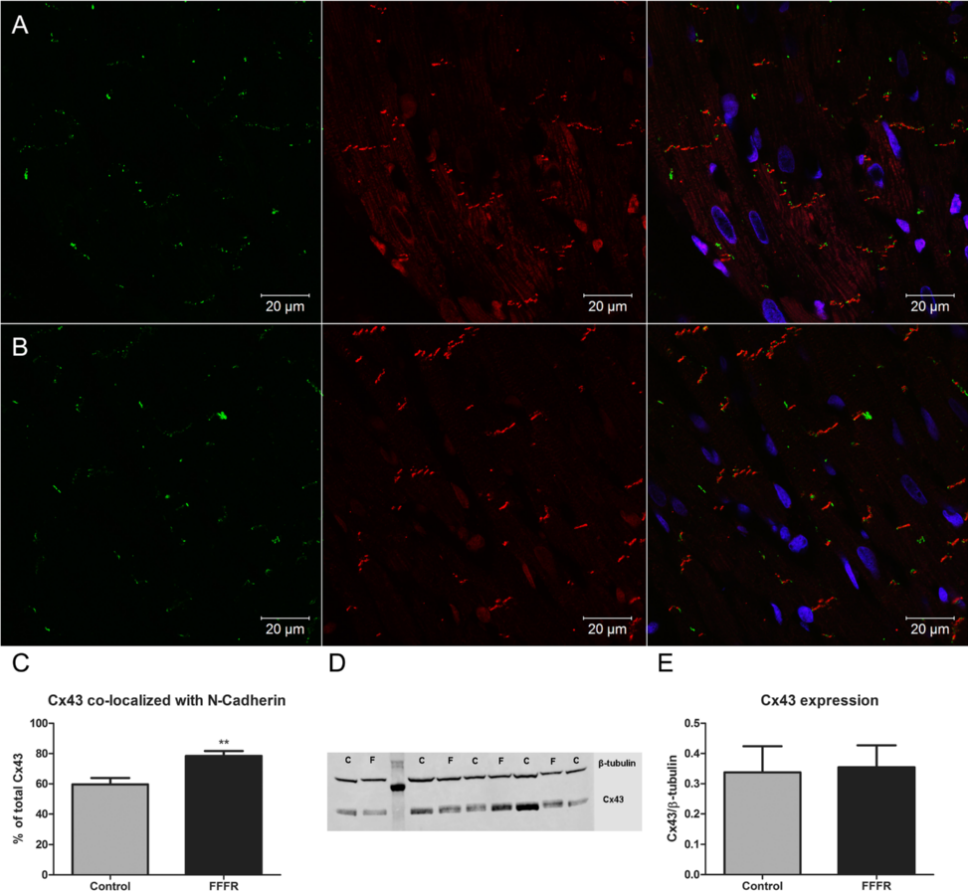
Connexin43 (Cx43) localization and expression. Representative immunofluorescence images of Cx43 (in green, left column), N-Cadherin (in red, middle column) and an overlay (right column) of Cx43 (green), N-Cadherin (*red*) and nuclei (*blue*). **a** Images from control hearts. **b** Images from fructose-fat fed rat (FFFR) hearts . The images shown are a maximum projection of 4 optical sections of heart tissue. **c** Quantification of Cx43 co-localized with N-Cadherin for control (*n* = 6) and FFFRs (*n* = 5). **d** Representative western blot of Cx43 and β-tubulin expression in cardiac tissue (C = control and F = FFFR). **e** Quantification of Cx43 expression (quantification was performed in duplicates and *n* = 4/group). Data are presented as mean ± SEM, ** *p* < 0.01

When evaluating the localization of Cx43 by immunohistochemistry (Fig. 7a-c), a higher fraction of Cx43 localized in approximation of N-cadherin was found in the FFFR group compared to the control group (78.4 ± 3.28 % (*n* = 5) vs. 59.6 ± 3.55 % (*n* = 6), *p* < 0.01). This indicates that a higher fraction of Cx43 is localized in gap junction plaques at the intercalated discs in the FFFRs compared to the controls. In contrast, we found that the expression level of the major ventricular gap junction protein Cx43 was similar between the two groups, when evaluated by western blotting (Fig. 7d and e). This is in accordance with the finding that gap junction conductance was unaffected between the two groups.

### Six weeks of high fructose-fat diet does not induce cardiac fibrosis

**Fig. 8:**
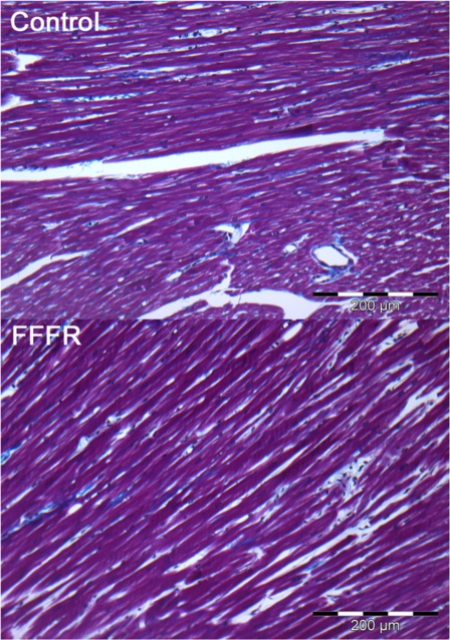
Cardiac fibrosis analysis. This figure shows representative images of Masson’s Trichrome stainings of the right ventricle from a control and a fructose-fat fed rat (FFFR). Masson’s trichrome stainings were used to show that there is no difference in fibrosis between the two groups

As seen in Fig. 8, there were no significant histological changes in cardiac tissue from either controls or FFFRs. The fraction of non-cardiomyocytes were 1.38 ± 0.36 % (*n* = 6) and 2.01 ± 0.26 % (*n* = 7), *p* = 0.17) for control and FFFR hearts respectively.

## Discussion

We have previously validated the FFFR model as a diet-induced rat model of metabolic syndrome [12]. Our previous study found no signs of contractile dysfunction under unstressed conditions in hearts from FFFRs for up to 48 weeks of feeding, but their hearts were more prone to asystole during reperfusion after ischemia [12]. This indicated electrical alterations in the heart of FFFRs. Abnormal electrical propagation, reflected as prolonged QRS and QT interval, as well as an increased risk of sudden cardiac death, is well established for type 2 diabetic patients (reviewed in [24]). The pathophysiological mechanism(s), however, are yet to be elucidated. Therefore, we investigated the electrophysiological characteristics of FFFRs, in the early pre-diabetic state, following 6 weeks of feeding.

In terms of metabolic status, we found that the FFFRs became overweight, with some hyperglycemia, hyperinsulinemia and increased plasma lipid levels, which was also reflected by increased intramyocardial triglyceride content. These findings, except for the hyperinsulinemia, are in agreement with our previous study [12], which also demonstrated that the FFFR model have a decreased intra venous glucose tolerance after 6 weeks of feeding. In our previous study, insulin levels were, however, not elevated until after 6 weeks of feeding. Nevertheless, together these data demonstrates that the FFFRs at 6 weeks of feeding can be characterized as a pre-diabetic model.

In the present study, we show that FFFRs display prolonged QRS and unchanged QT and QTc duration *in vivo*. An increased prevalence of QTc prolongation and dispersion is well established in diabetic patients [25, 26] and QT prolongation is also seen in diet-induced-obese (DIO) mice kept on a high-fat diet for 12–14 weeks [27]. The reason why we do not detect any QT changes in the FFFRs may simply be because our ECGs were recorded in the early pre-diabetic state after just 6 weeks of feeding; however, our QT data could also be influenced by the gradual downslope of the T-wave, which makes it difficult to detect the end of the QT interval in rodent ECGs. We do, however, detect a significant prolongation of the QRS complex in the FFFR hearts. A widening of the QRS complex is also seen at the onset of diabetes in ZDF rats [28] and at 2 and 7 months of age in the type 2 diabetic Goto-Kakizaki rat [29], as well as in type 2 diabetic humans [30]. Prolongation of the QRS complex reflects the presence of intraventricular conductance abnormalities, such as bundle-branch block, decreased ventricular conduction velocity and/or ventricular hypertrophy. With respect to the latter, our data show that heart weight is not significantly increased in FFFRs compared to controls after 6 weeks of feeding and we have previously established that cardiac hypertrophy is not present until 18 weeks of feeding [12]. In the present study, we find that conduction velocity is significantly slowed in tissue strips from the right ventricle of FFFRs compared to control rats. We have previously made the same observation in type 2 diabetic ZDF rats [11] and a similar reduction in conduction velocity is also described for STZ induced type 1 diabetic rats [7]. The present study is, however, the first to show conduction slowing in the heart of a diet-induced animal model.

Conductance disturbances such as decreased conduction velocity increase the risk of ventricular arrhythmias. *In vivo* we made the observation that one of the six FFFRs presented with PVCs in trigeminy with two sinus beats under unstressed conditions. In addition, Langendorff perfused hearts from FFFRs responded differently to 30 min of LAD occlusion compared to hearts from control fed rats. Hearts from FFFRs preserved a higher contractility during ischemia, which was seen as a higher DLVP and dP/dt max, even though the area at risk was similar between control and FFFR hearts. The increased contractility of FFFR hearts during ischemia is in agreement with our previous findings in hearts from FFFRs following 56 weeks of feeding [12]. In the previous study, we found that the FFFR group at 56 weeks of feeding was more prone to develop asystole during re-perfusion, and the present study shows that this is not the case at 6 weeks of feeding where FFFRs are more prone to VF during re-perfusion. The increased susceptibility to VF during reperfusion is not caused by increased infarct size. The unaffected infarct size is in contrast to earlier studies, where rats keep on a high-fat diet for 15 weeks [10] or a sucrose-condensated milk diet for 20 weeks [31] both develops increased infarct sizes after ischemia-reperfusion. This discrepancy may, however, be explained by the different feeding periods and regimes. Therefore, we conclude that pre-diabetic FFFRs have an increased susceptibility to ventricular arrhythmias following reperfusion, which is not explained by changes in infarct size.

In pathophysiological conditions such as cardiac ischemia and heart failure, decreased conduction velocity and increased arrhythmogenicity have been related to altered gap junction coupling. Compromised gap junction coupling may arise from: 1) decreased expression of the major ventricular gap junction protein Cx43; 2) closure or reduced conductance of the gap junction channels (e.g. by altered phosphorylation state); or 3) loss of gap junctional channels from the intercalated discs. In STZ induced type 1 diabetic rats, altered expression, localization and phosphorylation of Cx43 have all been suggested as the cause of impaired conduction; however, data are contradicting [6–9, 32]. In the ZDF rat, a decrease in the amount of Cx43 co-localized with N-cadherin at the intercalated discs was found [11]. In the present study, we found that the Cx43 expression was unaltered between controls and FFFRs and that the fraction of Cx43 co-localized with N-cadherin was actually increased in the FFFR group. The increase in Cx43 at the intercalated disc did, however, not translate into an altered gap junction conductance in isolated cardiomyocytes. Despite the lack of evidence for changes in gap junction coupling in the isolated cells, it would still be interesting to examine the phosphorylation state of Cx43 in the intact FFFR heart. However, given the complex nature of Cx43 phosphorylation (For review see Axelsen et al. 2013 [33]), it requires an extensive mass spectrometry based analysis, which is beyond the scope of this paper. Nevertheless, the lack of changes in gap junction conductance indicates that although cardiac gap junction remodeling may be evident in some diabetic models, it is unlikely to be the primary cause of conduction disturbances and increased arrhythmogenesis in the pre-diabetic FFFR model.

Other mechanisms that may contribute to decreased conduction velocity is a decrease in cardiomyocyte excitability due to altered Na^+^-channel activity or changes in cell size. Altered I_Na_ density and increased cell capacitance have previously been related to conduction disturbances in the heart of alloxan-induced diabetic rabbits [34]. However, we found no difference in Na^+^-channel activity between FFFRs and controls in isolated ventricular cardiomyocytes. Cell capacitance was also unchanged between groups, suggesting that the altered conduction velocity in FFFRs cannot be explained by a difference in cell size or Na^+^-channel activity.

Excitation is opposed by the hyperpolarizing action of I_K1_, which in principle could slow conduction, but no differences were observed between groups, excluding this possibility. On the other hand, several studies have shown that K^+^ currents, especially the transient outward K^+^ current (*I*_to_) is related to repolarization abnormalities in diabetes (reviewed in [35]) and may lead to increased risk of arrhythmia. Therefore, we also evaluated the K^+^ currents in cardiomyocytes from FFFRs and control rats, however, as for the Na^+^ currents, no significant differences were observed.

In addition to changes in gap junction coupling and the excitability of the individual cells, another parameter, which may affect conduction velocity in cardiac tissue, is electrical obstacles such as fibrosis (Reviewed in [36]). However, neither the FFFRs used in this study nor the ZDF rats, which were previously reported to have decreased conduction velocity [11], have any detectable increase in fibrosis (Fig. 6). Another parameter which is, however, found to be altered in both the FFFR and the ZDF model, is the marked intramyocardial lipid accumulation. Lipotoxicity due to ectopic lipid accumulation is a well-known phenomenon in diabetic cardiomyopathy (reviewed by [37]). Lipotoxicity has been connected to changes in cardiac energy status and lipoapoptosis [37], as well as development of altered cardiac signaling and mitochondrial remodeling [38]. Using a model study, it has been shown that altered mitochondrial function may contribute to the development of an arrhythmogenic substrate in the heart, but very little is known about its effects on the electrophysiological properties of the heart. Based on these observations, cardiac lipid accumulation and its effect on cardiac signaling and mitochondrial function becomes interesting topics for future electrophysiological studies and may potentially contribute to the conduction disturbances seen in the FFFR model.

Another pro-arrhythmia mechanism, which remains unexplored in this study, is remodeling of the autonomic nervous system. Adrenoreceptor stimulation is found to trigger arrhythmias originating in the sinoatrial node of diabetic db/db mice [39] and remodeling of the autonomic nervous system may conspire to increase cardiac mortality in diabetic patients.

## Conclusion

With the data presented in this paper we demonstrate that FFFRs have QRS prolongation *in vivo*, as well as decreased conduction velocity *ex vivo*. One FFFR showed frequent PVCs under unstressed conditions *in vivo* and isolated hearts from FFFRs had increased susceptibility to VF following cardiac ischemia-reperfusion. At the same time, we found no alterations in gap junctional coupling, Na^+^ or K^+^ current density, cell size, or fibrosis. This shows that the mechanism of conductance disturbances in the pre-diabetic heart differs from the mechanisms seen in cardiac ischemia and heart failure. Further studies are therefore needed in order to determine the pro-arrhythmic role of specific pre-diabetic related complications such as intramyocardial lipid accumulation, mitochondrial dysfunction, altered cardiac signaling and metabolism. Increased knowledge of the pro-arrhythmic mechanism in pre-diabetic and diabetic hearts remains important to improve the anti-arrhythmic treatment of diabetic patients and to decrease the risk of sudden cardiac death in diabetes.

## Abbreviations

Cx43: Connexin43
DLVP: Developed left ventricular pressure
FFFR: Fructose-fat fed rat
HR: Heart rate
LAD: Left anterior descending coronary artery
PFA: Paraformaldehyde
PVC: Premature ventricular complex
QTc: Corrected QT interval
SEM: Standard error of the mean
STZ: Streptozotocin
TLC: Thin-layer chromatography
VF: Ventricular fibrillation
VT: Ventricular tachycardia
WW: Wet weight
ZDF: Zucker diabetic fatty
